# The mitochondrial genome of the fringe-lipped frog-eating bat *Trachops coffini* and its phylogenetic position among new world leaf-nosed bats (Chiroptera: Phyllostomidae)

**DOI:** 10.1080/23802359.2025.2470835

**Published:** 2025-03-18

**Authors:** Jesús Antonio Rocamontes-Morales, Anahí Martínez-Cárdenas, Jorge Ortega, J. Antonio Baeza

**Affiliations:** ^a^ Departamento de Conservación de la Biodiversidad, Laboratorio Ecología Evolutiva y Conservación, ECOSUR-Villahermosa, Villahermosa, Mexico; ^b^ Laboratorio de Biología de la Conservación, Universidad Autónoma de Zacatecas, Unidad Académica de Ciencias Biológicas, Zacatecas, Mexico; ^c^ Departamento de Zoología, Escuela Nacional de Ciencias Biológicas, Posgrado en Ciencias Químico-Biológicas, Laboratorio de Bioconservación y Manejo, Instituto Politécnico Nacional, Mexico City, Mexico; ^d^ Department of Biological Sciences, Clemson University, Clemson, SC, USA; ^e^ Smithsonian Marine Station at Fort Pierce, Fort Pierce, FL, USA; ^f^ Departamento de Biología Marina, Facultad de Ciencias del Mar, Universidad Católica del Norte, Coquimbo, Chile

**Keywords:** Phylogeny, phylomitogenomics, mitogenome, selective pressure

## Abstract

The fringe-lipped frog-eating bat *Trachops coffini* inhabits tropical and semitropical rainforests from Mexico to Brazil. In this study, we sequenced and assembled the complete mitochondrial genome of *T. coffini*. The entire mitogenome is a circular molecule, 16,960 bp in total length that contains 13 protein-coding genes, two ribosomal RNA genes, 22 transfer RNA genes, and one D-loop or control region (CR). The overall base composition is A = 32.43%, G = 13.43%, C = 24.26%, and T = 29.87%, with A + T content = 62.31%. A phylogenetic tree reconstructed using the 13 PCGs that included 59 taxa recovered *T. coffini* as a taxon sister to a fully-supported clade containing the genera *Tonatia*, *Lophostoma*, and *Phyllostomus*.

## Introduction

Phyllostomidae, the second most species-rich bat family, harbors the fringe-lipped frog-eating bat, recently reclassified as *Trachops coffini* (formerly known as *T. cirrhosus*, Gray 1847), found in tropical and semi-tropical rainforests in Latin America (Cramer et al. 2001; Da Silva Fonseca et al. 2024). Distinguished by dermal projections on its lips, *T. coffini* is an opportunistic omnivore primarily consuming insects, notably coleopterans, while also hunting frogs (Jones et al. 2020; Camacho et al. 2022). Previous research centered on its diet, geographic distribution, and taxonomic history. Given limited genomic resources, our study presents the first detailed assembly and characterization of *T. coffini*’s mitochondrial genome, aiding in understanding its biology. Utilizing translated protein-coding genes (PCGs), we established the phylogenetic position of the genus *Trachops* within Phyllostomidae.

## Materials and methods

**Figure 1.**
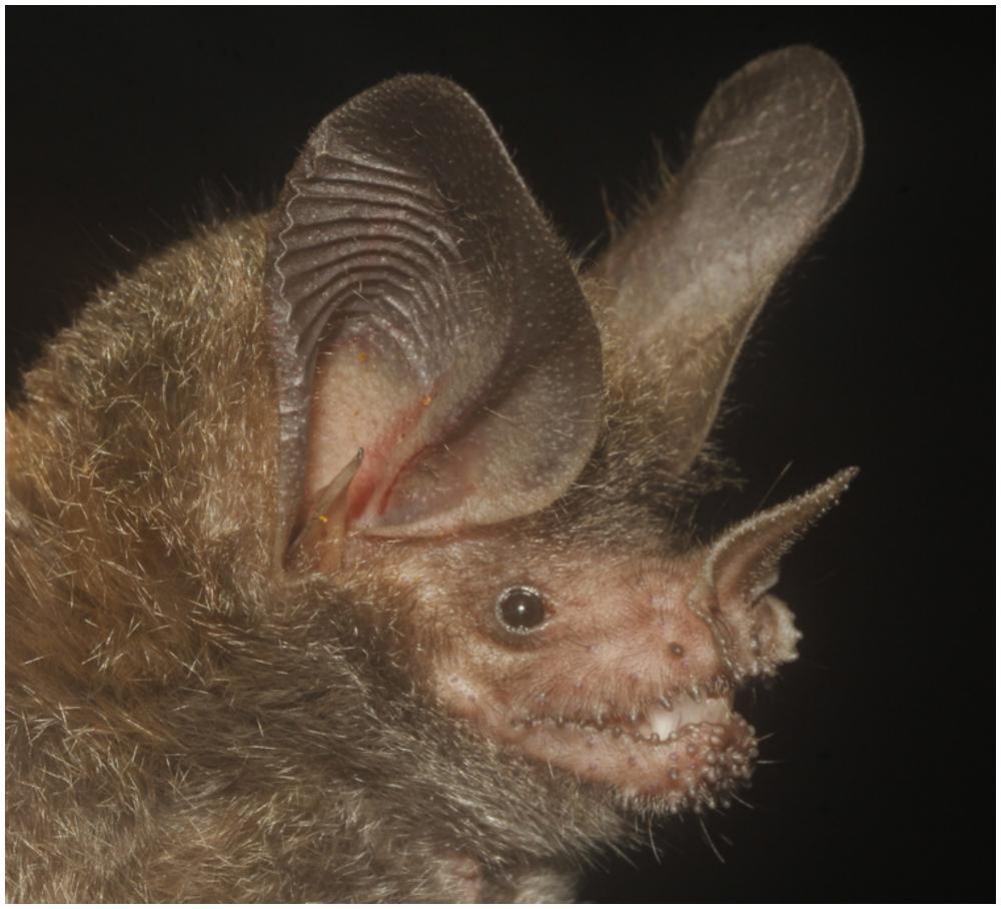
Image of *Trachops coffini.* The photo was taken by Juan Cruzado, used with permission.

Lung tissue from *T. coffini* (Figure 1) was collected in the archaeological zone of Palenque (Latitude** 17.484630°, Longitude** −92.046019°), Chiapas, México, in 2017 and preserved in RNAlater. The tissue was stored at the ENCB-IPN tissue bank (ID: ENCB_Chi-Phy_0629). Genomic DNA was extracted using the Quick-DNA™ HMW MagBead Kit (Zymo Research, Irvine, CA) protocol. DNA quality was assessed with a Nanodrop 2000 and quantified using a Qubit 4 fluorometer. A paired-end (PE, 150 bp) shotgun library was constructed and sequenced on an Illumina NovaSeq 6000 platform with a S4 flow cell at Novogene (Sacramento, CA). A total of 5,247,921,600 reads in FASTQ format were generated and utilized for *de novo* assembly of the mitochondrial genome.

The mitochondrial genome of *T. coffini* was *de novo* assembled using GetOrganelle v1.7.6.1 with a k-mer size of 115, using the mitochondrial genome (seed file) of *Tonatia saurophila* (GenBank: NC_022428.1) (Jin et al. 2020). We mapped the depth of coverage to ensure we obtained a high-quality genome (Supplementary Figure 1). Annotation was performed using the web server MITOS2 with the vertebrate genetic code (http://mitos2.bioinf.uni-leipzig.de/index.py – Donath et al. 2019). Nucleotide composition was estimated using MEGA v11.0.13 (Tamura et al. 2021). We visualized the circular genome using the Proksee web server (https://proksee.ca/ – Grant et al. 2023). Codon usage of PCGs was determined using the vertebrate mitochondrial code on the Codon Usage web server, while amino acid frequencies and Relative Synonym Codon Usage (RSCU) were calculated using the EZcodon tool on the EZmito web server (http://ezmito.unisi.it/ezcodon – Cucini et al. 2021). Secondary structures of mitochondrial tRNA genes were predicted using MiTFi and visualized with FORNA (Jühling et al. 2012; Kerpedjiev et al. 2015).

Selective constraints in each mitochondrial PCG were explored by estimating the number of nonsynonymous substitutions per nonsynonymous site (KA) and synonymous substitutions per synonymous site (KS) using KaKs_calculator 2.0. The KA/KS ratio (*ω*) was calculated for each gene, with values indicating neutrality (*ω* = 1), negative or purifying selection (*ω* < 1), and positive or diversifying selection (*ω* > 1), based on pairwise comparisons with *Tonatia saurophila*. The γ-MYN model was applied during calculations to adjust for variable mutation rates along the sequences (Wang et al. 2009).

Examination of the control region (CR) included calculating nucleotide composition in MEGA v11.0.13 and detecting microsatellites (short sequence repeats, SSRs) using Microsatellite Repeats Finder (Bikandi et al. 2004). Tandem repeats were identified using Tandem Repeats Finder (Benson 1999). The secondary structure of this region was predicted using FORNA.

The phylogenetic position of *T. coffini* within Phyllostomidae was investigated by analyzing its assembled genome along with mitogenomes we verified from 59 other related species available in GenBank. Outgroup terminals comprised species from five families.

We employed the MitoPhAST v3.0 pipeline for maximum-likelihood (ML) phylogenetic reconstruction (Tan et al., 2015). The concatenated and partitioned PCG amino acid alignment with the 13 PCGs was then used to construct a ML phylogenetic tree using IQ-TREE (Nguyen et al. 2015). The robustness of the tree topology was assessed by 1000 bootstrap pseudo-replicates.

All software resources used in this work are declared in Supplementary Table 1.

## Results and discussion

**Figure 2.**
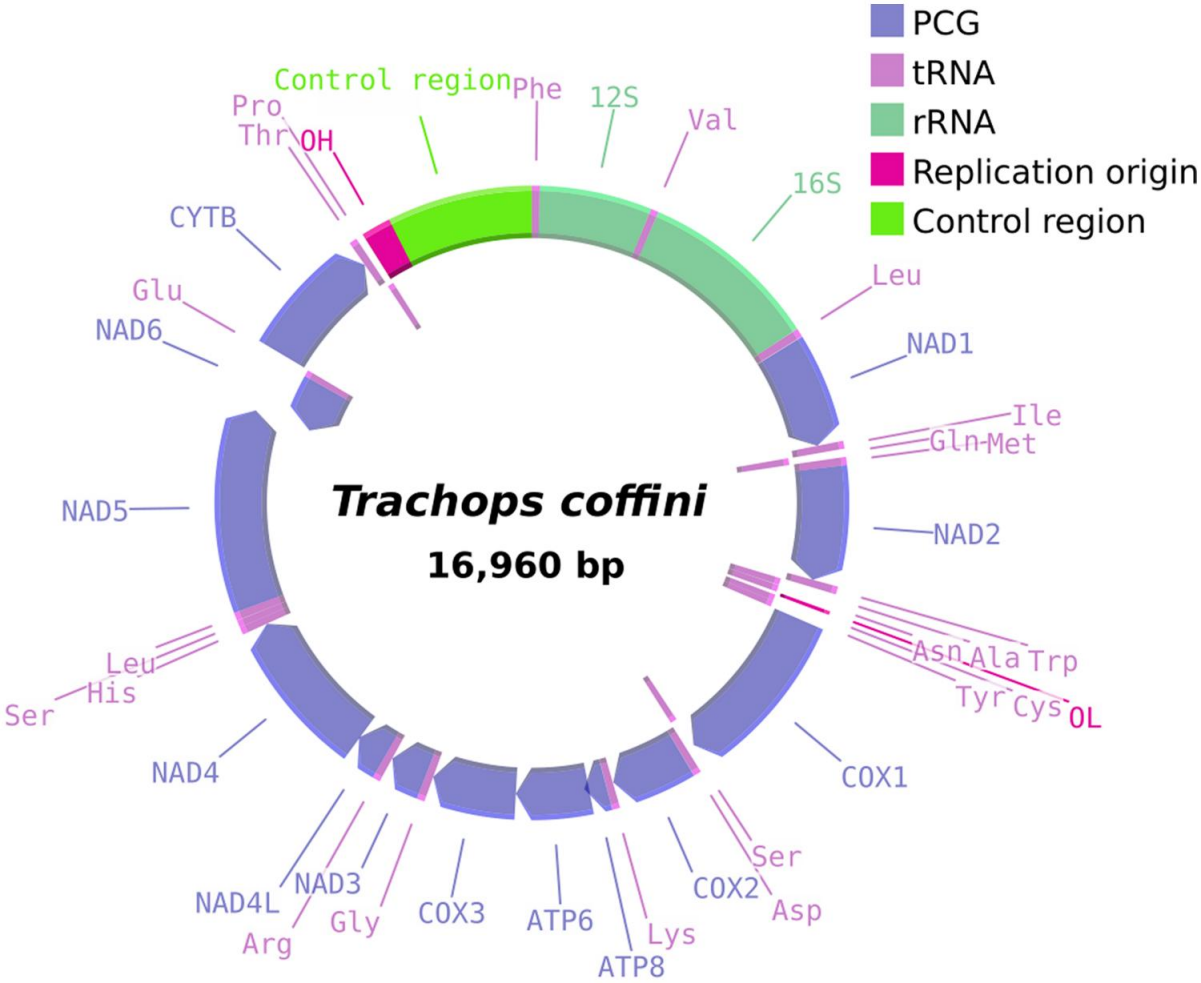
Circular depiction of the mitochondrial genome belonging to *Trachops coffini*. Colors indicate gene composition and arrangement in the mitochondrial genome. The origin of replication of the light (OL) and heavy (OH) chains is annotated in the figure.

The mitogenome (GenBank: PP271916) of the fringe-lipped frog-eating bat *T. coffini* is 16,960 bp in length and encodes 37 genes; 13 PCGs, 22 tRNA genes, and two rRNA (rrnS and rrnL) genes, and a relatively long non-coding region 1515 bp long (Figure 2).

Most PCG genes and tRNAs are encoded on the heavy (H) strand, while only one PCG (NAD6) and eight tRNAs (trnQ, trnA, trnN, trnC, trnY, trnS2, trnE, and trnP) are encoded in the light strand (L) (Table 1). Gene composition and arrangement in the mitochondrial genome are identical to those previously reported for phyllostomids (Supplementary Table 2).

**Table 1.** Characteristics of the mitochondrial genome of *Trachops coffini.*

Feature	Type	Start codon	Stop codon	Strand	Length (bp)	Start codon	Stop codon	Anticodon	Continuity
Phe	tRNA	1	70	+	70			GAA	0
12S rRNA	rRNA	71	1041	+	971				0
Val	tRNA	1042	1109	+	68			TAC	0
16S rRNA	rRNA	1110	2673	+	1564				1
Leu	tRNA	2675	2749	+	75			TAA	2
NAD1	PCG	2752	3708	+	957	ATG	TAA		−1
Ile	tRNA	3708	3776	+	69			GAT	−3
Gln	tRNA	3774	3846	–	73			TTG	0
Met	tRNA	3847	3916	+	70			CAT	0
NAD2	PCG	3917	4963	+	1047	ATT	TAG		−2
Trp	tRNA	4962	5030	+	69			TCA	5
Ala	tRNA	5036	5105	–	70			TGC	2
Asn	tRNA	5108	5180	–	73			GTT	2
OL		5183	5214	+	32			GCA	−1
Cys	tRNA	5214	5279	–	66				0
Tyr	tRNA	5280	5346	–	67			GT	1
COX1	PCG	5348	6892	+	1545	ATG	TAA		−3
Ser	tRNA	6890	6960	–	71			TGA	5
Asp	tRNA	6966	7032	+	67			GTC	1
COX2	PCG	7034	7717	+	684	ATG	TAA		3
Lys	tRNA	7721	7787	+	67			TTT	1
ATP8	PCG	7789	7992	+	204	ATG	TAA		−43
ATP6	PCG	7950	8630	+	681	ATG	TAA		−1
COX3	PCG	8630	9414	+	785	ATG	T(AA)		−1
Gly	tRNA	9414	9482	+	69			TCC	0
NAD3	PCG	9483	9830	+	348	ATT	TAA		0
Arg	tRNA	9831	9898	+	68			TCG	0
ND4l	PCG	9899	10195	+	297	ATG	TAA		−7
ND4	PCG	10189	11566	+	1378	ATG	T(AA)		0
His	tRNA	11567	11634	+	68			GTG	0
Ser	tRNA	11635	11694	+	60			GCT	1
Leu	tRNA	11696	11765	+	70			TAG	0
NAD5	PCG	11766	13595	+	1830	ATA	TAA		−26
NAD6	PCG	13570	14097	–	528	ATG	TAA		0
Glu	tRNA	14098	14165	–	68			TTC	4
CYTB	PCG	14170	15309	+	1140	ATG	AGA		0
Thr	tRNA	15310	15378	+	69			TGT	−1
Pro	tRNA	15378	15444	–	67			TGG	256
OH		15701	16016	+	316				944
Control region		15445	16960	+	1515				

Stop codons that are shown with parentheses to indicate ambiguous or incomplete codons.

The nucleotide composition of the positive DNA strand of the mitochondrial genome in *T. coffini* was as follows: A = 32.43%, G = 13.43%, C = 24.26%, and T = 29.87%, resulting in a G + C content of 37.69% and an A + T content of 62.31%, consistent with the range observed in other phyllostomids (Supplementary Table 2).

In *Trachops coffini*’s PCGs, each amino acid is encoded by at least two different codons (Supplementary Table 3), with a preference for codons ending with adenine over those ending with thymine and cytosine, while codons ending with guanine are the least utilized. Relative synonymous codon usage (RSCU) and amino acid frequency were summarized (Supplementary Figures 2 and 3). The most frequently used codons are CTA (Leu), ATA (Met), and ATT (Ile), while CGG (Arg) and GCG (Ala) are the least frequently used codons. Similar observations have been reported in other phyllostomids (Supplementary Table 2).

Analysis of KA/KS ratios for all mitochondrial PCGs (Supplementary Figure 4 and Table 4) revealed values <1 (all *p* values <.05), indicating purifying selection across all genes. CYTB (COB), COX1, COX2, and COX3 exhibited the lowest KA/KS values, while ATP8 followed by NAD6 showed the greatest values. The average KA/KS ratio for the 13 PCGs was 0.0619 (±0.0777). These findings align with observations in other phyllostomids (Barrera et al. 2023), highlighting the importance of structural conservation in mitochondrial-encoded genes (Supplementary Table 2).

**Figure 3.**
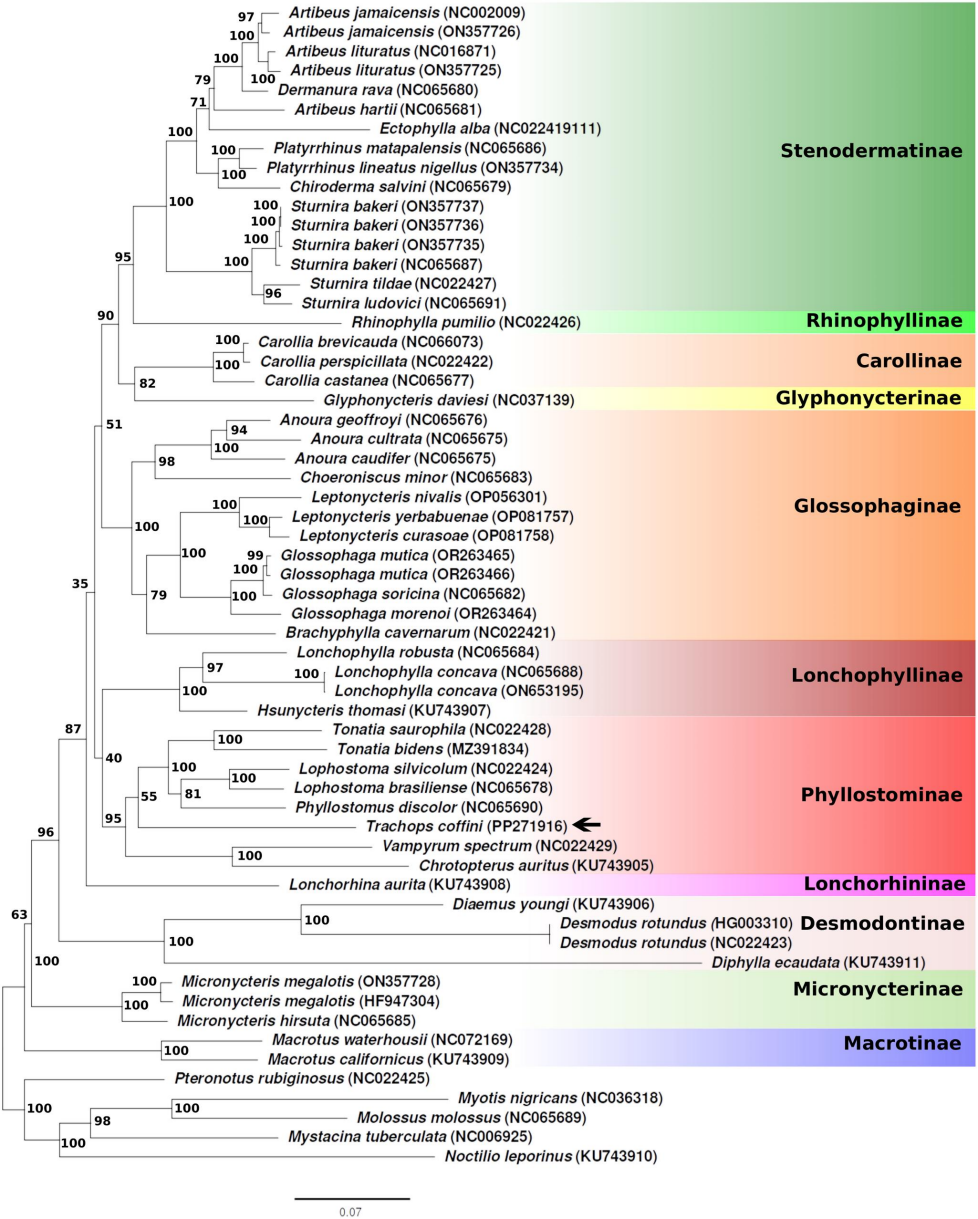
Phylogenetic ML analysis of the family Phyllostomidae, based on mitochondrial genomes, including the species assembled for this study: *Trachops coffini* (marked with an arrow on the tree). The numbers displayed above/below nodes represent the bootstrap values, indicating the level of support for the depicted branches.

In our ML phylogenetic analysis (Figure 3), the tree topology fullly supported the monophyly of the Phyllostomidae (bootstrap value [bv] = 100), consistent with previous studies based on mitochondrial PCGs (Supplementary Table 2). Within Phyllostomidae, the subfamily Phyllostominae received robust support (bv = 95). *T. coffini* appeared as a weakly supported sister taxon (bv = 55) to a fully-supported clade comprising the genera *Tonatia*, *Lophostoma*, and *Phyllostomus*. The tree topology, particularly the position of *T. coffini*, agrees with previously proposed phylogenetic relationships among phyllostomids utilizing complete genomes, and relationships based on morphological characters of *T. coffini* (Wetterer et al. 2000; Botero-Castro et al. 2013; Botero-Castro et al. 2018; Camacho et al. 2022; Barrera et al. 2023; Vargas-Trejo et al. 2023).

## Conclusions

In this report, we assembled and annotated the complete mitochondrial genome of *T. coffini.* A phylogenetic tree was reconstructed based on all translated PCGs and demonstrated that *T. coffini* groups as sister taxon to the genera *Tonatia*, *Lophostoma*, and *Phyllostomus*. The information of the present study will provide valuable data for further genetic studies on *Trachops*. To further clarify the evolutionary relationships within the genus, additional genomic data should be included in future studies to test the monophyly of *Trachops*.

## Supplementary Material

Supplemental Material

## Data Availability

Mitochondrial genome sequence can be accessed via accession number PP271916 in GenBank of NCBI. The associated BioProject, SRA, and Bio-Sample numbers are PRJNA1021227, SRR27951645, and SAMN39926795, respectively. The tissue for *Trachops coffini* was stored at the ENCB-IPN tissue bank (ID: ENCB_Chi-Phy_0629) in charge of Jorge Ortega (artibeus2@aol.com).
