# Supplementary material for: The mitochondrial genome of the fringe-lipped frog-eating bat Trachops coffini and its phylogenetic position among new world leaf-nosed bats (Chiroptera: Phyllostomidae)
Source: Mitochondrial DNA B Resour. 2025 Mar 18;10(4):298–303. doi: 10.1080/23802359.2025.2470835 (PMC11921155; doi:10.1080/23802359.2025.2470835)
Supplement: Supplemental Material [file TMDN_A_2470835_SM0178.docx]

**Supplementary Material**

**Detailed description of Control Region, tRNA structures, and ribosomal genes**

The Control Region (CR, Supplementary Figure 6) in *T. coffini* spans 1,523 bp with an AT content of 55.4%, like other phyllostomids (Supplementary Table 2). Microsatellite Repeat Finder identified 3 di-nucleotide microsatellites, 3 mononucleotide microsatellites, one Tandem Repeat motif and three conserved domains were identified in the CR (Supplementary Table 5 and Supplementary Table 6). These domains, from 5′ to 3′, include Extended Termination Associated Sequences (ETAS) with conserved motifs ETAS-1 and ETAS-2, central domains (CD) with conserved motifs B to F, and Conserved Sequence Blocks (CSB) with motifs CSB-1 to -3, hypothesized to prime H-strand replication.

All tRNA genes exhibit anticodon, acceptor, DHU, and TΨC stems (Supplementary Figure 5) and display a cloverleaf secondary structure, except for tRNA-Serine 1, which lacks the DHU arm. The length and structural features of these tRNA genes resemble those observed in other species, including phyllostomid bats (Supplementary Table 2).

The rrnS (12S) and rrnL (16S) genes in *T. coffini’s* mitochondrial genome are 971 bp and 1,564 bp long, respectively. The rrnS gene is positioned between tRNAF and tRNAV, while rrnL is located between tRNAV and tRNAL2 (Table 1). These genes exhibit an AT composition of 59% (12S) and 62% (16S).

**Tables and Figures**

Supplementary Table 1. Software resources for *T. coffini* mitochondrial genome analysis.

| **Resource** | **Author** | **Link** |
| --- | --- | --- |
| Codon Usage | Storhatd, 2000 | http://www.bioinformatics.org/sms2/codon_usage.html |
| EZcodon | Lee, 2018 | http://ezmito.unisi.it/ezcodon |
| FORNA | Kerpedijiev et al. 2015 | http://rna.tbi.univie.ac.at/forna |
| GenomeVX | Conant & Wolfe, 2008 | http://wolfe.ucd.ie/GenomeVx |
| GetOrganelle v 1.7.6.1 | Jin et al. 2020 | https://github.com/Kinggerm/GetOrganelle/releases |
| IQ-TREE | Nguyen et al. 2015 | http://www.iqtree.org/ |
| KaKs_calculator 2.0 | Wang et al. 2015 | https://github.com/kullrich/kakscalculator2 |
| MEGA v 11.0.13 | Tamura et al. 2021 | https://www.megasoftware.net/ |
| Microsatellite Repeats Finder | Bikandi et al. 2004 | http://insilico.ehu.es/mini_tools/microsatellites |
| MiTFi | Jüling et al. 2012 | https://doi.org/10.1093/nar/gkr1131 |
| MitoPhast | Tan et al. 2015 | https://github.com/mht85/MitoPhAST |
| MITOS2 | Donath et al. 2019 | http://mitos2.bioinf.uni-leipzig.de/index.py |
| Proksee | Grant et al. 2023 | https://proksee.ca/ |
| Tandem Repeats Finder | Benson, 1999 | https://tandem.bu.edu/trf/trf.html |

Supplementary Table 2. Nucleotide Composition from Phyllostomidae Family mitochondrial genomes. The report from this study is highlighted in bold.

| **Species** | **Genbank** | **Mitogenome** | | | | **Control Region** | | | | **Subfamily** | **Reference** | **DOI** |
| --- | --- | --- | --- | --- | --- | --- | --- | --- | --- | --- | --- | --- |
|  |  | **T** | **C** | **A** | **G** | **T** | **C** | **A** | **G** |  |  |  |
| *A. jamaicencis* | NC 002009.1 | 30 | 25 | 32 | 13 | 26 | 26 | 31 | 17 | Stenodermatinae | Pumo et al., 1998 | 10.1007/pl00006430 |
| *A. lituratus* | NC 016871.1 | 30 | 25 | 32 | 13 | 26 | 27 | 31 | 16 | Stenodermatinae | Pumo et al., 1998 | [10.1007/pl00006430](https://doi.org/10.1007/pl00006430) |
| *A. hartii* | NC 065681.1 | 29 | 25 | 32 | 13 | 29 | 27 | 28 | 16 | Stenodermatinae | Pumo et al., 1998 | [10.1007/pl00006430](https://doi.org/10.1007/pl00006430) |
| *C. salvini* | NC 065679.1 | 27 | 28 | 32 | 13 | 25 | 28 | 30 | 17 | Stenodermatinae | Camacho et al., 2022 | [10.1093/zoolinnean/zlac055](https://doi.org/10.1093/zoolinnean/zlac055) |
| *D. rava* | NC 065680.1 | 28 | 26 | 32 | 13 | 26 | 26 | 31 | 16 | Stenodermatinae | Camacho et al., 2022 | [10.1093/zoolinnean/zlac055](https://doi.org/10.1093/zoolinnean/zlac055) |
| *E. alba* | NC 041639.1 | 29 | 25 | 33 | 13 | 26 | 27 | 30 | 16 | Stenodermatinae | Vivas-Toro et al., | [10.1016/j.gene.2021.145868](https://doi.org/10.1016/j.gene.2021.145868) |
| *P. matapalensis* | NC 065686.1 | 27 | 28 | 32 | 13 | 29 | 25 | 31 | 15 | Stenodermatinae | Camacho et al., 2022 | [10.1093/zoolinnean/zlac055](https://doi.org/10.1093/zoolinnean/zlac055) |
| *S. bakeri* | NC 065687.1 | 25 | 30 | 32 | 13 | 26 | 26 | 31 | 17 | Stenodermatinae | Camacho et al., 2022 | [10.1093/zoolinnean/zlac055](https://doi.org/10.1093/zoolinnean/zlac055) |
| *S. ludovici* | NC 065691.1 | 25 | 30 | 32 | 13 | 25 | 28 | 31 | 17 | Stenodermatinae | Camacho et al., 2022 | [10.1093/zoolinnean/zlac055](https://doi.org/10.1093/zoolinnean/zlac055) |
| *S. tildae* | NC 022427.1 | 25 | 30 | 32 | 13 | 24 | 29 | 32 | 15 | Stenodermatinae | Botero-Castro et al., 2013 | [10.1016/j.ympev.2013.07.003](https://doi.org/10.1016/j.ympev.2013.07.003) |
| *S. parvidens* | MW554922 | 26 | 29 | 31 | 15 | 25 | 28 | 31 | 17 | Stenodermatinae | Baeza et al., 2021 | 10.1093/jmammal/gyab117 |
| *R. pumilio* | NC 022426.1 | 29 | 25 | 32 | 14 | 28 | 23 | 28 | 20 | Rhinophyllinae | Botero-Castro et al., 2013 | [10.1016/j.ympev.2013.07.003](https://doi.org/10.1016/j.ympev.2013.07.003) |
| *C. brevicauda* | NC 066073.1 | 27 | 28 | 31 | 14 | 26 | 28 | 29 | 17 | Carollinae | Camacho et al., 2022 | [10.1093/zoolinnean/zlac055](https://doi.org/10.1093/zoolinnean/zlac055) |
| *C. castanea* | NC 065677.1 | 27 | 29 | 31 | 14 | 27 | 28 | 28 | 17 | Carollinae | Camacho et al., 2022 | [10.1093/zoolinnean/zlac055](https://doi.org/10.1093/zoolinnean/zlac055) |
| *C. perspicillata* | NC 022422.1 | 27 | 28 | 31 | 14 | 26 | 28 | 28 | 17 | Carollinae | Botero-Castro et al., 2013 | [10.1016/j.ympev.2013.07.003](https://doi.org/10.1016/j.ympev.2013.07.003) |
| *C. auritus* | NC 037132.1 | 25 | 30 | 29 | 16 | 27 | 25 | 30 | 18 | Carollinae | Botero-Castro et al., 2013 | [10.1016/j.ympev.2013.07.003](https://doi.org/10.1016/j.ympev.2013.07.003) |
| *L. brasiliense* | NC 065678.1 | 27 | 28 | 32 | 13 | 27 | 26 | 31 | 16 | Phyllostominae | Camacho et al., 2022 | [10.1093/zoolinnean/zlac055](https://doi.org/10.1093/zoolinnean/zlac055) |
| *L. silvicolum* | NC 022424.1 | 26 | 29 | 32 | 13 | 29 | 25 | 30 | 16 | Phyllostominae | Botero-Castro et al., 2013 | [10.1016/j.ympev.2013.07.003](https://doi.org/10.1016/j.ympev.2013.07.003) |
| *L. concava* | NC 065688.1 | 29 | 26 | 31 | 14 | 31 | 23 | 27 | 19 | Phyllostominae | Camacho et al., 2022 | [10.1093/zoolinnean/zlac055](https://doi.org/10.1093/zoolinnean/zlac055) |
| *L. robusta* | NC 065684.1 | 26 | 29 | 31 | 15 | 29 | 24 | 28 | 19 | Phyllostominae | Camacho et al., 2022 | [10.1093/zoolinnean/zlac055](https://doi.org/10.1093/zoolinnean/zlac055) |
| *M. californicus* | NC_037136.1 | 28 | 26 | 32 | 14 | 27 | 27 | 29 | 17 | Macrotinae | Vargas-Trejo et al., 2023 | 10.1016/j.gene.2023.147295 |
| *M. waterhousii* | NC_072169.1 | 28 | 27 | 32 | 13 | 26 | 26 | 31 | 17 | Macrotinae | Vargas-Trejo et al., 2023 | 10.1016/j.gene.2023.147295 |
| *M. hirsuta* | NC 065685.1 | 25 | 29 | 32 | 14 | 25 | 29 | 29 | 17 | Micronycterinae | Camacho et al., 2022 | [10.1093/zoolinnean/zlac055](https://doi.org/10.1093/zoolinnean/zlac055) |
| *M. megalotis* | NC 022419.1 | 25 | 29 | 32 | 14 | 27 | 27 | 30 | 16 | Micronycterinae | Botero-Castro et al., 2013 | [10.1016/j.ympev.2013.07.003](https://doi.org/10.1016/j.ympev.2013.07.003) |
| *P. discolor* | NC 065690.1 | 27 | 28 | 31 | 14 | 27 | 28 | 28 | 17 | Phyllostominae | Camacho et al., 2022 | [10.1093/zoolinnean/zlac055](https://doi.org/10.1093/zoolinnean/zlac055) |
| *T. coffini* | PP271916 | 30 | 24 | 32 | 13 | 27 | 27 | 28 | 18 | Phyllostominae | Present report | Present report |
| *T. saurophila* | NC 022428.1 | 26 | 27 | 34 | 13 | 29 | 25 | 30 | 16 | Phyllostominae | Botero-Castro et al., 2013 | [10.1016/j.ympev.2013.07.003](https://doi.org/10.1016/j.ympev.2013.07.003) |
| *D. rotundus* | NC 022423.1 | 23 | 32 | 30 | 16 | 27 | 24 | 29 | 20 | Desmodontinae | Botero-Castro et al., 2013 | [10.1016/j.ympev.2013.07.003](https://doi.org/10.1016/j.ympev.2013.07.003) |
| *D. youngi* | NC 037133.1 | 25 | 31 | 31 | 14 | 26 | 27 | 30 | 17 | Desmodontinae | Botero-Castro et al., 2013 | [10.1016/j.ympev.2013.07.003](https://doi.org/10.1016/j.ympev.2013.07.003) |
| *D. ecaudata* | NC 037138.1 | 25 | 29 | 33 | 13 | 28 | 25 | 32 | 15 | Desmodontinae | Botero-Castro et al., 2013 | [10.1016/j.ympev.2013.07.003](https://doi.org/10.1016/j.ympev.2013.07.003) |
| *A. caudifer* | NC_022420.1 | 28 | 26 | 32 | 14 | 28 | 25 | 29 | 18 | Glossophaginae | Botero-Castro et al., 2013 | [10.1016/j.ympev.2013.07.003](https://doi.org/10.1016/j.ympev.2013.07.003) |
| *A. cultrata* | NC 065675.1 | 29 | 25 | 32 | 13 | 30 | 23 | 31 | 17 | Glossophaginae | Camacho et al., 2022 | [10.1093/zoolinnean/zlac055](https://doi.org/10.1093/zoolinnean/zlac055) |
| *A. geoffroyi* | NC 065676.1 | 27 | 27 | 32 | 13 | 28 | 24 | 31 | 17 | Glossophaginae | Camacho et al., 2022 | [10.1093/zoolinnean/zlac055](https://doi.org/10.1093/zoolinnean/zlac055) |
| *C. minor* | NC 065683.1 | 29 | 25 | 33 | 13 | 26 | 27 | 30 | 17 | Glossophaginae | Camacho et al., 2022 | [10.1093/zoolinnean/zlac055](https://doi.org/10.1093/zoolinnean/zlac055) |
| *G. soricina* | NC 065682.1 | 31 | 23 | 32 | 14 | 30 | 23 | 31 | 16 | Glossophaginae | Camacho et al., 2022 | [10.1093/zoolinnean/zlac055](https://doi.org/10.1093/zoolinnean/zlac055) |
| *L. curasoae* | NC_066832.1 | 27 | 27 | 31 | 15 | 30 | 23 | 28 | 19 | Glossophaginae | Barrera et al., 2023 | 10.1016/j.gene.2023.147588 |
| *L. nivalis* | NC_066830.1 | 28 | 26 | 31 | 15 | 29 | 24 | 29 | 17 | Glossophaginae | Barrera et al., 2023 | 10.1016/j.gene.2023.147588 |
| *L. yerbabuenae* | NC_066831.1 | 27 | 27 | 31 | 15 | 29 | 23 | 29 | 19 | Glossophaginae | Barrera et al., 2023 | 10.1016/j.gene.2023.147588 |
| *M. harrisoni* | ON260904.1 | 28 | 26 | 33 | 13 | No reported | | | | Glossophaginae | Basaldúa et al., 2023 | [10.1080/00222933.2022.2150581](https://doi.org/10.1080/00222933.2022.2150581) |
| *B. cavernarum* | NC 022421.1 | 27 | 27 | 32 | 14 | 28 | 26 | 29 | 18 | Glossophaginae | Botero-Castro et al., 2013 | [10.1016/j.ympev.2013.07.003](https://doi.org/10.1016/j.ympev.2013.07.003) |

Supplementary Table 3. Codon usage analysis of PCGs in the mitochondrial genome of *T. coffini*. AA= Amino Acid.

| **AA** | **Codon** | **Number** | **/1000** | **Fraction** | **AA** | **Codon** | **Number** | **/1000** | **Fraction** |
| --- | --- | --- | --- | --- | --- | --- | --- | --- | --- |
| Ala | GCG | 3 | 0.79 | 0.01 | Pro | CCG | 5 | 1.31 | 0.02 |
|  | GCA | 86 | 22.59 | 0.36 |  | CCA | 73 | 19.18 | 0.36 |
|  | GCT | 84 | 22.06 | 0.35 |  | CCT | 64 | 16.81 | 0.32 |
|  | GCC | 66 | 17.34 | 0.28 |  | CCC | 60 | 15.76 | 0.3 |
| Cys | TGT | 13 | 3.41 | 0.48 | Gln | CAG | 5 | 1.31 | 0.06 |
|  | TGC | 14 | 3.68 | 0.52 |  | CAA | 81 | 21.28 | 0.94 |
| Asp | GAT | 40 | 10.51 | 0.61 | Arg | CGG | 2 | 0.53 | 0.03 |
|  | GAC | 26 | 6.83 | 0.39 |  | CGA | 39 | 10.24 | 0.61 |
| Glu | GAG | 13 | 3.41 | 0.14 |  | CGT | 13 | 3.41 | 0.2 |
|  | GAA | 81 | 21.28 | 0.86 |  | CGC | 10 | 2.63 | 0.16 |
| Phe | TTT | 139 | 36.51 | 0.61 | Ser | AGT | 28 | 7.35 | 0.1 |
|  | TTC | 88 | 23.12 | 0.39 |  | AGC | 30 | 7.88 | 0.11 |
| Gly | GGG | 24 | 6.3 | 0.11 |  | TCG | 4 | 1.05 | 0.01 |
|  | GGA | 100 | 26.27 | 0.45 |  | TCA | 74 | 19.44 | 0.26 |
|  | GGT | 53 | 13.92 | 0.24 |  | TCT | 86 | 22.59 | 0.3 |
|  | GGC | 44 | 11.56 | 0.2 |  | TCC | 60 | 15.76 | 0.21 |
| His | CAT | 56 | 14.71 | 0.59 | Thr | ACG | 13 | 3.41 | 0.04 |
|  | CAC | 39 | 10.24 | 0.41 |  | ACA | 143 | 37.56 | 0.44 |
| Ile | ATT | 204 | 53.59 | 0.61 |  | ACT | 104 | 27.32 | 0.32 |
|  | ATC | 129 | 33.88 | 0.39 |  | ACC | 68 | 17.86 | 0.21 |
| Lys | AAG | 11 | 2.89 | 0.12 | Val | GTG | 10 | 2.63 | 0.05 |
|  | AAA | 78 | 20.49 | 0.88 |  | GTA | 101 | 26.53 | 0.55 |
| Leu | TTG | 14 | 3.68 | 0.02 |  | GTT | 53 | 13.92 | 0.29 |
|  | TTA | 139 | 36.51 | 0.23 |  | GTC | 20 | 5.25 | 0.11 |
|  | CTG | 31 | 8.14 | 0.05 | Trp | TGG | 7 | 1.84 | 0.07 |
|  | CTA | 253 | 66.46 | 0.42 |  | TGA | 98 | 25.74 | 0.93 |
|  | CTT | 112 | 29.42 | 0.18 | Tyr | TAT | 76 | 19.96 | 0.56 |
|  | CTC | 59 | 15.5 | 0.1 |  | TAC | 60 | 15.76 | 0.44 |
| Met | ATG | 38 | 9.98 | 0.15 | End | AGG | 0 | 0 | 0 |
|  | ATA | 218 | 57.26 | 0.85 |  | AGA | 1 | 0.26 | 0.09 |
| Asn | AAT | 83 | 21.8 | 0.54 |  | TAG | 1 | 0.26 | 0.09 |
|  | AAC | 71 | 18.65 | 0.46 |  | TAA | 9 | 2.36 | 0.82 |

Supplementary Table 4. Ka/Ks from PCGs of *Trachops coffini* vs *Tonatia saurophila*. S-site = synonymous sites; N-site = non-synonymous.

| **Sequence** | **Ka** | **Ks** | **Ka/Ks** | **P-Value** | **Length** | **S-Sites** | **N-Sites** |
| --- | --- | --- | --- | --- | --- | --- | --- |
| ATP6 | 0.0364498 | 1.37698 | 0.026471 | 6.34E-59 | 678 | 161.443 | 516.557 |
| ATP8 | 0.176896 | 0.678108 | 0.260866 | 0.0006145 | 201 | 59.1191 | 141.881 |
| COX1 | 0.0139741 | 1.42856 | 0.009782 | 7.70E-160 | 1542 | 380.578 | 1161.42 |
| COX2 | 0.0452692 | 3.79721 | 0.011922 | 1.14E-78 | 681 | 158.055 | 522.945 |
| COX3 | 0.0114006 | 2.65323 | 0.004297 | 3.80E-93 | 783 | 163.419 | 619.581 |
| CYTB | 0.0402235 | 3.84239 | 0.010468 | 3.30E-83 | 1137 | 278.896 | 858.104 |
| ND1 | 0.0407655 | 2.20736 | 0.018468 | 1.59E-70 | 954 | 249.569 | 704.431 |
| ND2 | 0.0960451 | 1.96005 | 0.049001 | 3.33E-52 | 1041 | 241.044 | 799.956 |
| ND3 | 0.0978366 | 3.12873 | 0.031270 | 1.38E-18 | 345 | 77.3842 | 267.616 |
| ND4 | 0.0790742 | 1.88833 | 0.041875 | 1.59E-83 | 1377 | 366.486 | 1010.51 |
| ND4L | 0.0652985 | 1.06472 | 0.061329 | 1.87E-20 | 294 | 84.3498 | 209.65 |
| ND5 | 0.105687 | 1.60474 | 0.065859 | 7.39E-89 | 1806 | 475.75 | 1330.25 |
| ND6 | 0.160768 | 0.751107 | 0.214041 | 3.43E-11 | 525 | 142.166 | 382.834 |

Supplementary Table 5. Microsatellites sequence found in the CR mitochondrial genome of *T. coffini*.

| **Position** | **Cicle** | **Repeats** | **Sequence** |
| --- | --- | --- | --- |
| 55 | 2 | 3 | TATATA |
| 214 | 2 | 3 | ATATAT |
| 1222 | 2 | 3 | GCGCGC |
| 1261 | 2 | 3 | CCCCCC |
| 1270 | 2 | 3 | CCCCCC |
| 1454 | 2 | 3 | AGAGAG |
| 1480 | 2 | 4 | TTTTTTTT |

Supplementary Table 6. Microsatellites sequence found in the CR mitochondrial genome of *T. coffini*.

| **Position** | **Length** | **Ps** | **Cn** | **A** | **C** | **G** | **T** | **Motif** |
| --- | --- | --- | --- | --- | --- | --- | --- | --- |
| 789--1194 | 406 | 14 | 28.8 | 21 | 35 | 21 | 21 | CACCTGTACGTACG |


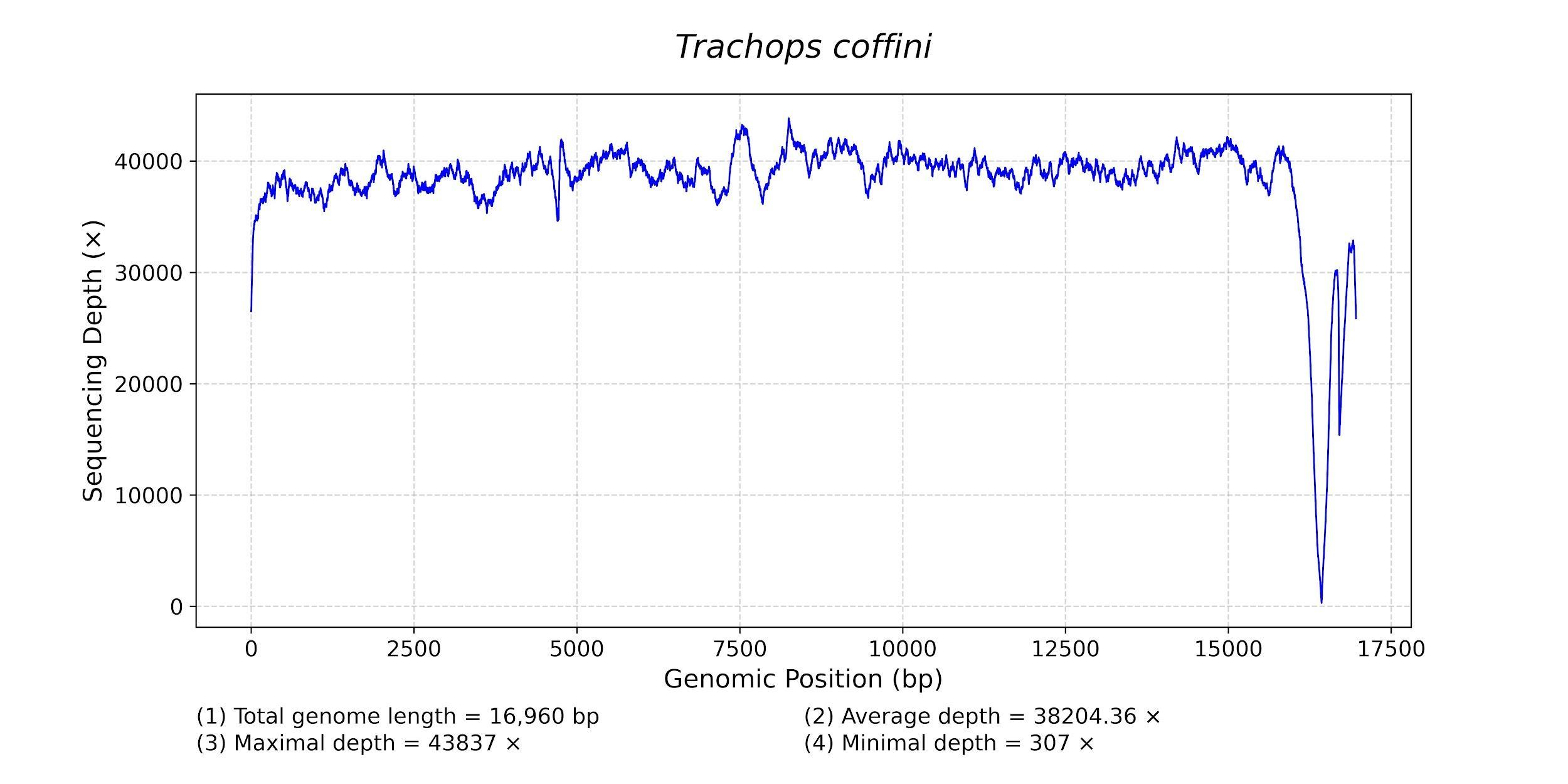


Supplementary Figure 1. Depth of coverage for *Trachops coffini* mitochondrial genome.


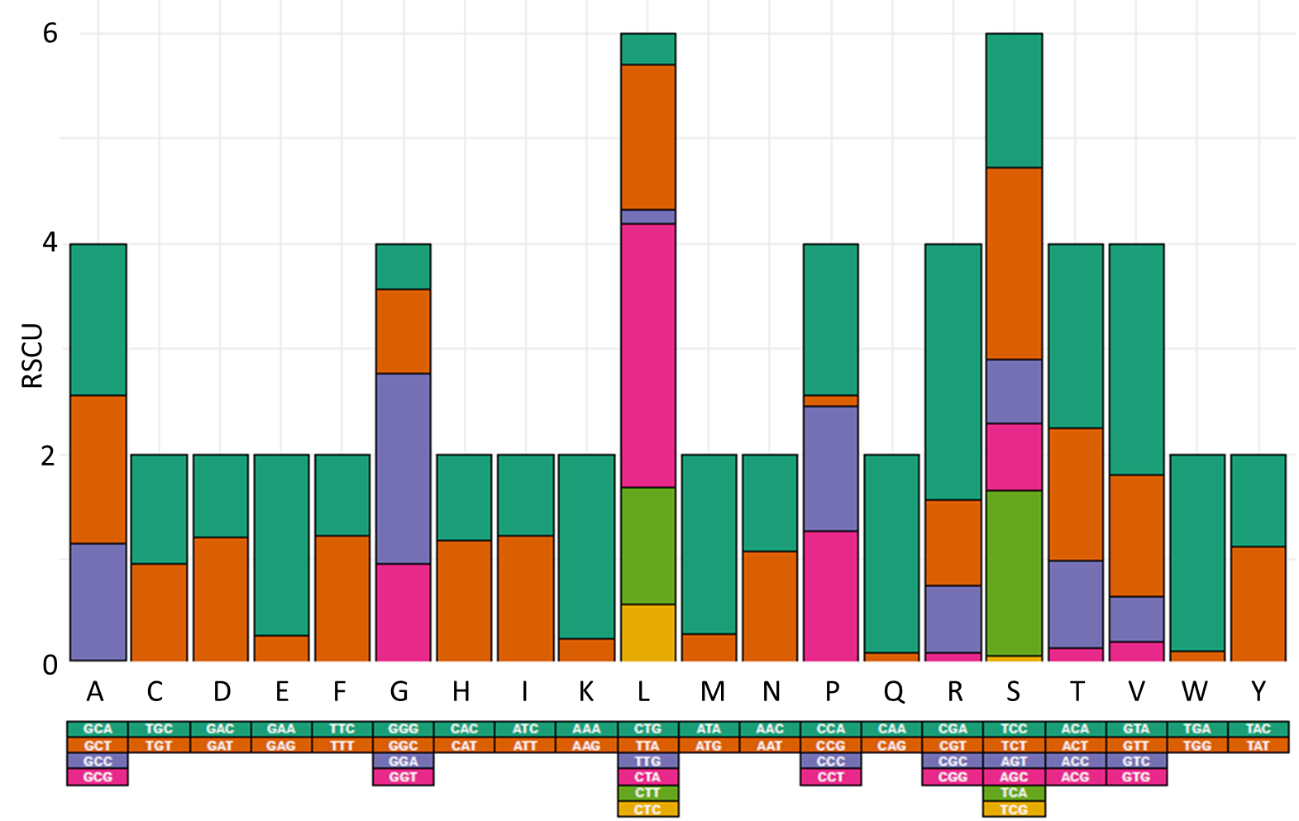


Supplementary Figure 2. Relative Synonymous Codon Usage (RSCU) for the PCG’s of *T. coffini*, performed with EZcodon web tool (Cucini et al., 2021).


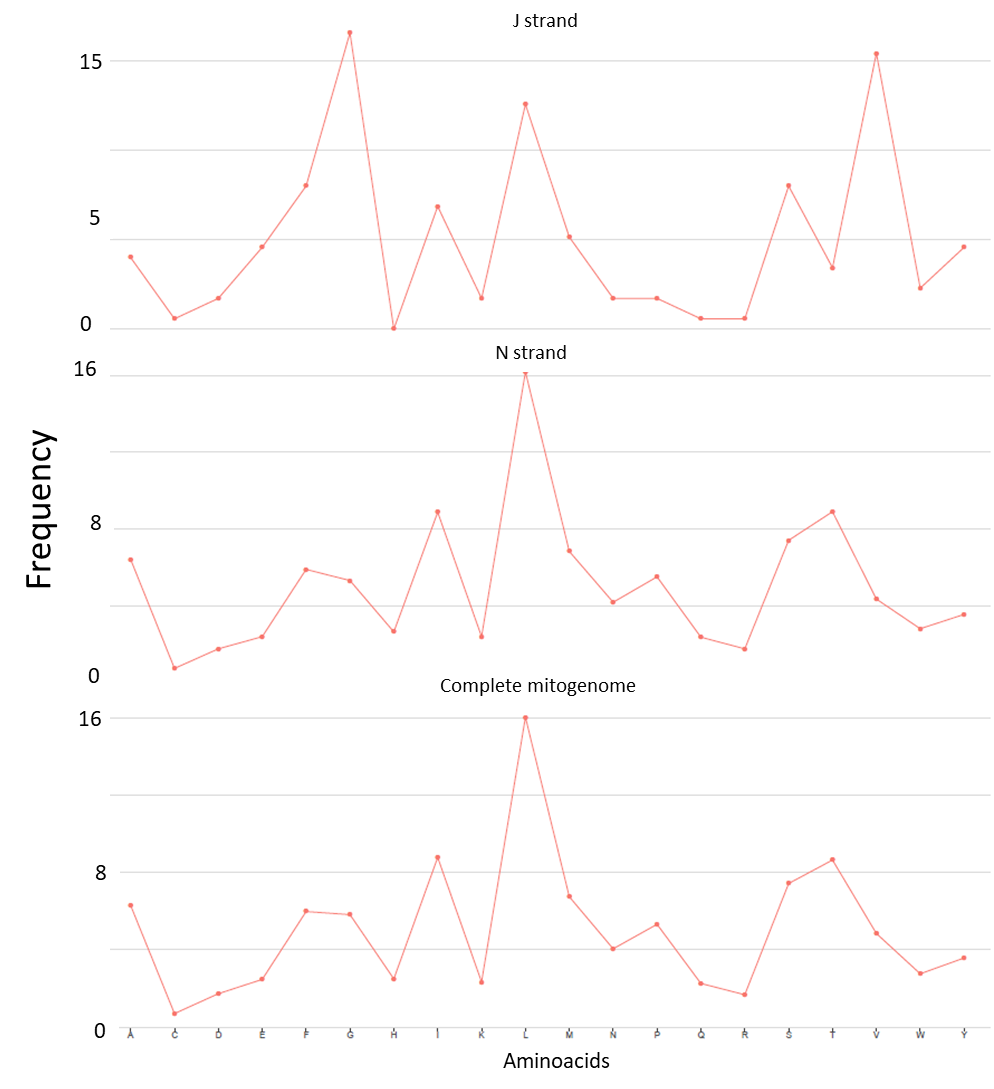


Supplementary Figure 3. Amino acid frequency in the mitochondrial genome of *T. coffini*. J and N strand represents Light and Heavy chain on circular mitogenome.


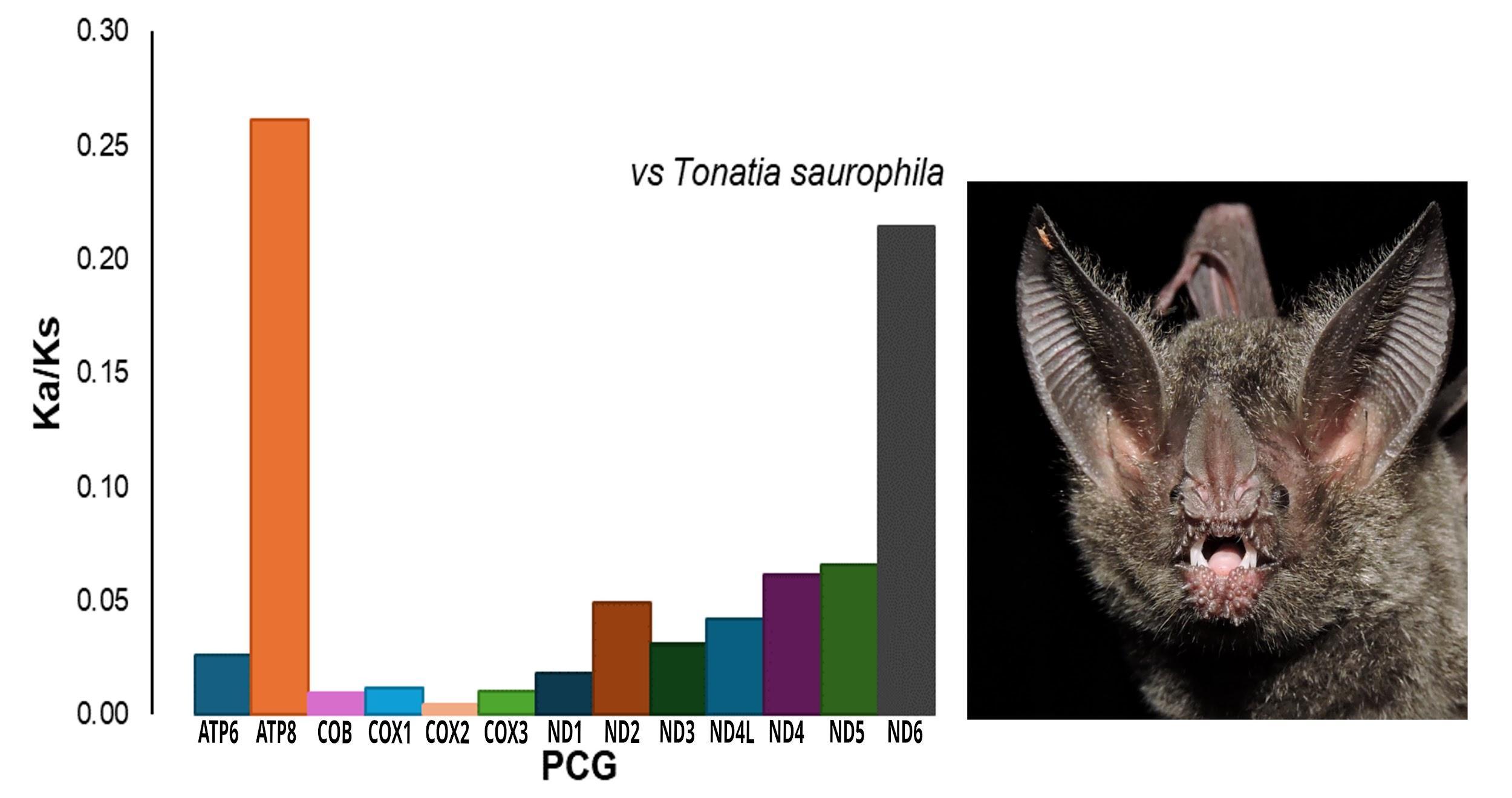


Supplementary Figure 4. Selective pressure analysis in the PCGs in *T.coffini*. Ka/Ks ratio for each PCG was calculated using *Tonatia saurophila* as outgroups (Supplementary Table 2). Image of *T. coffini* from Juan Cruzado, used with permission.


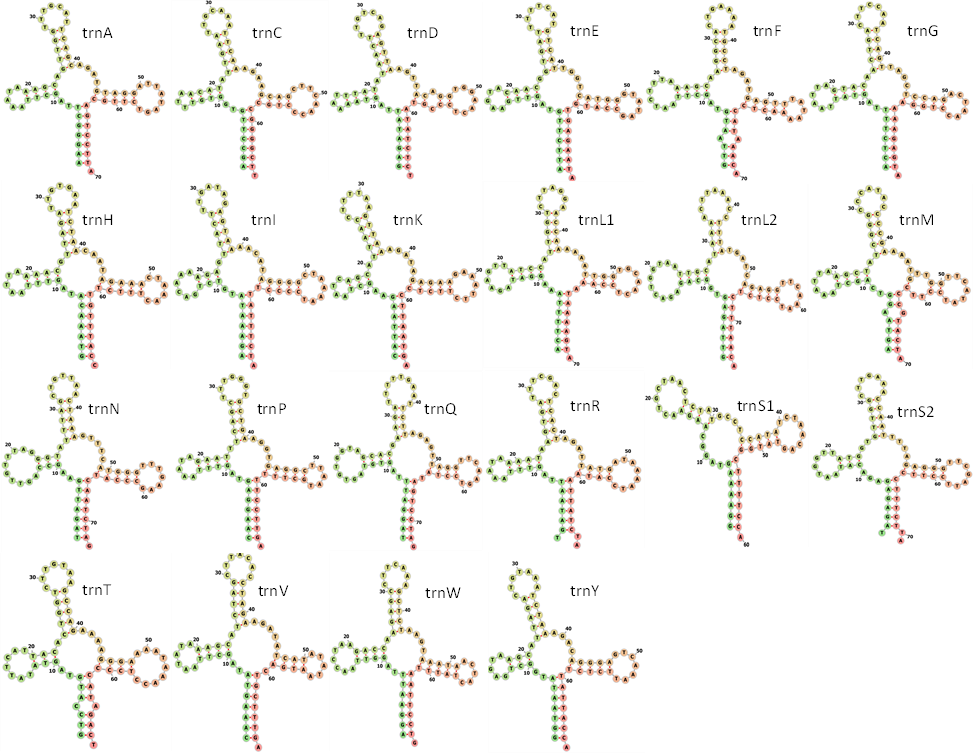


Supplementary Figure 5. Secondary structure of the 22 tRNAs in the mitochondrial genome of *T. coffini*.


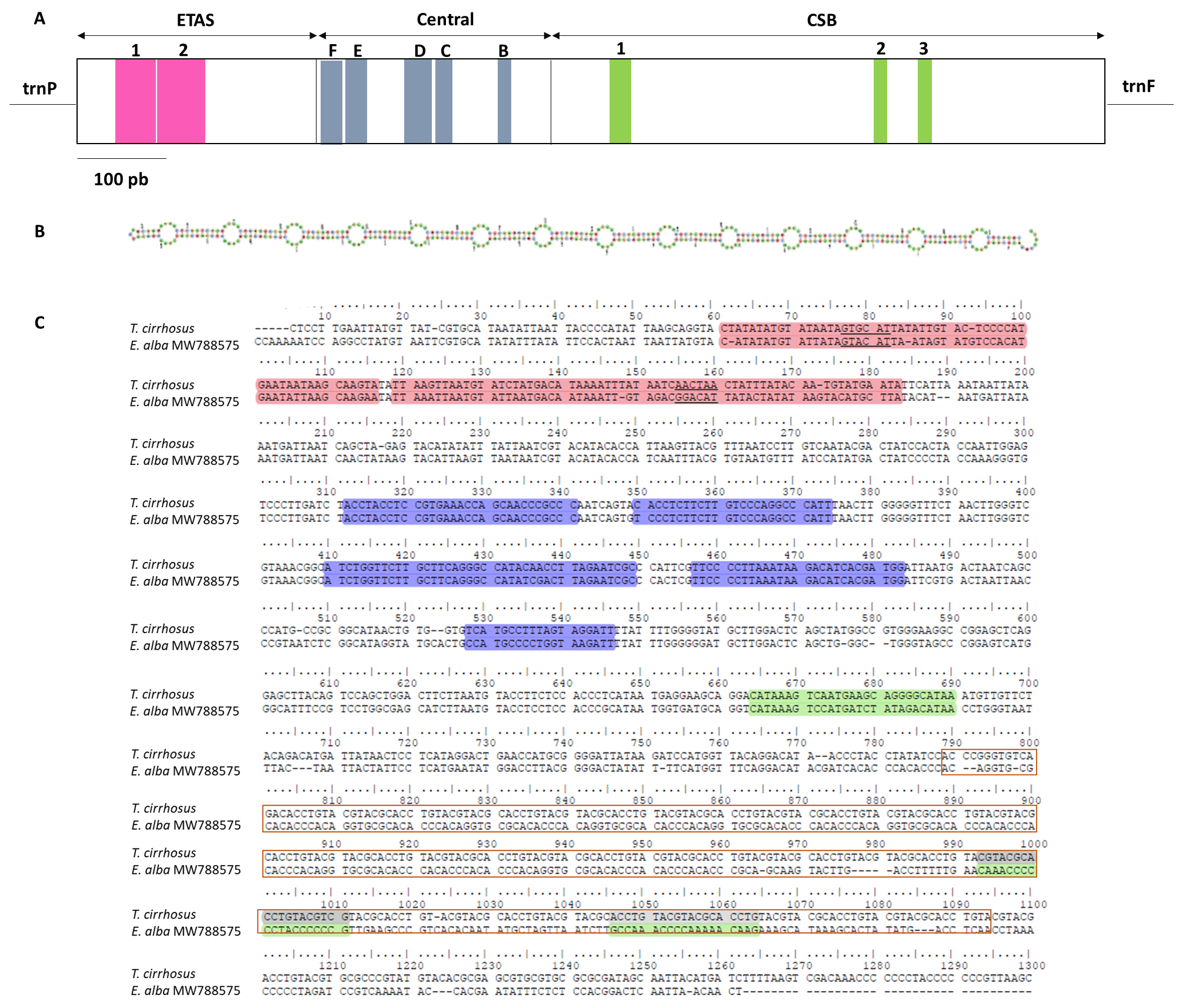


Supplementary Figure 6. Mitochondrial control region (CR) sequence of *Trachops coffini* aligned to *E. alba*. A) The CR is separated into an extended termination associated sequence (ETAS) domain, a central domain, and a conserved sequence block (CSB) domain. Locations of the conserved ETA 1 and ETA 2 blocks within the ETAS domain, conserved boxes F, E, D, C, and B within the central domain, and CSB1, CSB2, and CSB3 boxes within the CSB domain are shown. B) Secondary structure of tandem repeats, a long hairpin with internal loops (pos. 789-1194). C) Domains and features in the mitochondrial CR. The conserved blocks within each domain are highlighted. Tandem repeat region is highlighted in the red box.
